# Tissue-Nonspecific Alkaline Phosphatase, a Possible Mediator of Cell Maturation: Towards a New Paradigm

**DOI:** 10.3390/cells10123338

**Published:** 2021-11-28

**Authors:** Masahiro Sato, Issei Saitoh, Yuki Kiyokawa, Yoko Iwase, Naoko Kubota, Natsumi Ibano, Hirofumi Noguchi, Youichi Yamasaki, Emi Inada

**Affiliations:** 1 Department of Genome Medicine, National Center for Child Health and Development, 2-10-1 Okura, Setagaya, Tokyo 157-8535, Japan; sato-masa@ncchd.go.jp; 2 Department of Pediatric Dentistry, Asahi University School of Dentistry, Gifu 501-0296, Japan; isaitoh@dent.asahi-u.ac.jp (I.S.); ykiyokawa@dent.asahi-u.ac.jp (Y.K.); bano@dent.asahi-u.ac.jp (N.I.); 3 Department of Dentistry for the Disabled, Asahi University School of Dentistry, Gifu 501-0296, Japan; iwase@dent.asahi-u.ac.jp; 4 Department of Pediatric Dentistry, Graduate School of Medical and Dental Sciences, Kagoshima University, Kagoshima 890-8544, Japan; k1744111@dent.kagoshima-u.ac.jp (N.K.); yamasaki@dent.kagoshima-u.ac.jp (Y.Y.); 5 Department of Regenerative Medicine, Graduate School of Medicine, University of the Ryukyus, Okinawa 903-0215, Japan; noguchih@med.u-ryukyu.ac.jp

**Keywords:** alkaline phosphatase, tissue-nonspecific alkaline phosphatase, pluripotent stem cells, somatic stem cells, signal regulator, juvenile cells, reprogramming, induced pluripotent stem cells

## Abstract

Alkaline phosphatase (ALP) is a ubiquitous membrane-bound glycoprotein capable of providing inorganic phosphate by catalyzing the hydrolysis of organic phosphate esters, or removing inorganic pyrophosphate that inhibits calcification. In humans, four forms of ALP cDNA have been cloned, among which tissue-nonspecific ALP (TNSALP) (TNSALP) is widely distributed in the liver, bone, and kidney, making it an important marker in clinical and basic research. Interestingly, TNSALP is highly expressed in juvenile cells, such as pluripotent stem cells (i.e., embryonic stem cells and induced pluripotent stem cells (iPSCs)) and somatic stem cells (i.e., neuronal stem cells and bone marrow mesenchymal stem cells). Hypophosphatasia is a genetic disorder causing defects in bone and tooth development as well as neurogenesis. Mutations in the gene coding for TNSALP are thought to be responsible for the abnormalities, suggesting the essential role of TNSALP in these events. Moreover, a reverse-genetics-based study using mice revealed that TNSALP is important in bone and tooth development as well as neurogenesis. However, little is known about the role of TNSALP in the maintenance and differentiation of juvenile cells. Recently, it was reported that cells enriched with TNSALP are more easily reprogrammed into iPSCs than those with less TNSALP. Furthermore, in bone marrow stem cells, ALP could function as a “signal regulator” deciding the fate of these cells. In this review, we summarize the properties of ALP and the background of ALP gene analysis and its manipulation, with a special focus on the potential role of TNSALP in the generation (and possibly maintenance) of juvenile cells.

## 1. General Property of Alkaline Phosphatase (ALP)

Alkaline phosphatase (ALP; EC 3.1.3.1) is a ubiquitous membrane-bound glycoprotein found in many organisms, from bacteria to humans. In most cases, they are homodimeric enzymes, and each catalytic site contains three metal ions, i.e., two Zn and one Mg. The enzymes catalyze the hydrolysis by catalyzing the hydrolysis of organic phosphate esters, or removing inorganic pyrophosphate (PPi), an inhibitor of biomineralization [1]. As physiological substrates for ALPs, PPi, phosphoethanolamine (PE), and pyridoxal 5′-phosphate (PLP) are generally known [1]. For example, ALP (which is specifically known as tissue-nonspecific alkaline phosphatase (TNSALP), as shown later) isolated from human SAOS-2 osteosarcoma cells hydrolyzes PE and PLP at physiologic pH [2]. ALPs also appear to be involved in the metabolism of nucleotides. For instance, Say et al. [3] reported that purified TNSALP could hydrolyze adenosine triphosphate (ATP), adenosine diphosphate (ADP), adenosine monophosphate (AMP), PPi, glucose-1-phosphate, glucose-6-phosphate, fructose-6-phosphate, β-glycerophosphate, bis-(p-nitrophenyl)-phosphate and p-nitrophenyl phosphate.

ALP was originally described in a histochemical study as a marker for various tissues, especially bone and tooth formation-related tissues [4]. Additionally, ALP is reportedly highly expressed in juvenile cells, such as early preimplantation mouse embryos (cleavage stage embryos and inner cell mass (ICMs)) [5,6], pluripotent stem cells (PSCs)/embryonic stem cells (ESCs)/induced pluripotent stem cells (iPSCs) [7,8], primordial germ cells (PGCs) [9] and spermatogonia [10], stem cells for spermatogenesis, and some types of somatic stem cells, such as neuronal stem cells (NSCs) [11], and bone marrow mesenchymal stem cells (BMMSCs) [12]. Moreover, ALP is known to be highly expressed in the intestine, placenta, adipose, B lymphocytes, colon T lymphocytes, and osteoblasts (precursors for bone formation) [13]. Besides the normal cells, ALP expression is correlated with the progression of some types of cancers (i.e., colon cancer, osteosarcomas, neuroblastoma, and leukemia), making it a clinical marker for these cancer cells [13].

Clinically, a mutation in the gene coding for TNSALP has been closely associated with a severe skeletal deformity disease termed “hypophosphatasia (HPP),” which is characterized by several pathological abnormalities, including rickets, osteomalacia, epilepsy-like seizures associated with vitamin B6 deficiency, muscle weakness, and respiratory disturbance [14,15].

These findings suggest that ALP has some roles in various biological systems. 

## 2. ALP Isoforms and Their Detailed Properties 

In humans, at least four forms of *ALP* cDNA have been cloned: intestinal ALP (IALP or IAP; restricted to the intestine), placental ALP (PLALP, PLAP or Regan isozyme; restricted to the placenta), germ cell ALP (GCALP, GCAP or NAGAO isozyme; restricted to early embryonic cells), and liver/bone/kidney ALP (L/B/K ALP; widely distributed) [1,16]. The last form is generally called TNSALP or TNAP [1,16]. Similarly, at least four forms of ALP (i.e., embryonic ALP (EAP), IALP, a putative pseudogene, and TNSALP) have been identified in mice [1,16]. In humans, the gene for TNSALP is located on chromosome 1, and the genes for the other three isoforms (IALP, PLALP, and GCALP) are located on chromosome 2 [16].

Notably, PLALP and GCALP have approximately 98% homology, PLALP and IALP have approximately 87% homology, and IALP and TNSALP have only approximately 57% homology [17]. According to Whyte [18], the gene encoding TNSALP seems to be the ancestral gene. PLALP, GCALP, IALP, and TNSALP belong to the ALP family.

### 2.1. PLALP

PLALP is a polymorphic heat-stable enzyme present at high levels in the placenta, with up to 18 allozymes resulting from point mutations, in contrast to the other ALP isoenzymes [16]. The gene coding for PLALP is defined as *ALPP* (Table 1) [17], and the gene can be re-expressed by cancer cells as the Regan isoenzyme [16].

The biological functions of PLALP have been extensively determined by reverse genetics-based analysis. For example, Skynner et al. [20] demonstrated that systemic overexpression of human PLALP has no adverse effects on mouse development or viability using transgenic (Tg) mouse lines. Based on these findings, they suggested that PLALP could be used as a reporter gene in conjunction with, or as an alternative to ß-galactosidase (ß-gal; encoded by *lacZ*). A similar conclusion was also drawn by DePrimo et al. [21] (Table 2). The results of knock-out (KO), knock-in (KI), and Tg mouse models for assessing the gene function of the ALP family are summarized in Table 2.

### 2.2. IALP 

IALP is a partially heat-stable isozyme present at high levels in intestinal tissue. In contrast to the other ALP isoenzymes, the carbohydrate side-chains of IALP are not terminated by sialic acid [16].

Although trace expression of IALP has been detected in the thymus, IALP expression in mice and humans is largely restricted to the gut during late embryonic development and adult stages [37]. Notably, IALP can be re-expressed in cancer cells as a modified form, designated as Kasahara isoenzyme [16,38]. The gene encoding IALP is defined as *ALP1* in humans [17] (Table 1) and Akp3 in mice (Table 1) [1,17], which encodes duodenum-specific intestinal IALP (dIALP) [19]. Notably, Akp6 encodes global IALP (gIALP) [19].

To examine the biological functions of IALP, Narisawa et al. [22] generated *Akp3* null-mutated mice, called *Akp3^−/−^* mice, which were histologically normal and fertile. However, long-term maintenance on a high-fat diet resulted in higher body weight gain compared with wild-type (WT) animals. Histological examination revealed the accelerated transport of fat droplets through the intestinal epithelium and elevated serum triglyceride levels in the *Akp3^−/−^* mice compared to that in WT mice. Narisawa et al. [22] suggested that IALP participates in a rate-limiting step, regulating fat absorption (Table 2).

Notably, Lynes et al. [39] demonstrated that efficient fat (long-chain fatty acid, LCFA) transport across the small intestinal epithelium is mediated by IALPs and the putative fatty acid translocase/CD36. According to them, phosphorylated CD36 (pCD36) exists in mouse enterocytes, and pCD36 is a substrate of gIALP. gIALP-mediated dephosphorylation results in increased LCFA transport capability.

### 2.3. GCALP

GCALP is a heat-stable isozyme present at low levels in germ cells, embryonal, and some neoplastic tissues [16]. Similar to IALP, it can be re-expressed as a NAGAO isozyme in cancer cells [16]. The gene encoding GCALP is defined as *ALPP2* (Table 1) [1,17].

To examine the properties of the *ALPP2* promoter, Narisawa et al. [24] generated several Tg mouse lines harboring the entire human *ALPP2* gene. The results demonstrated that the 450 bp promoter sequence directed human *ALPP2* expression to the intestine and endothelial cells, whereas the 1.7 kb promoter sequence directed human *ALPP2* expression to the spermatogenic lineage and to the eight-cell embryos through the blastocysts (Table 2). More importantly, *ALPP2* expression in Tg mice induced cellular immune tolerance to GCALP. Narisawa et al. [24] suggested that these *ALPP2* Tg mice could be useful for studying immune responses associated with repeated administration of conjugated or derivatized anti-GCALP monoclonal antibodies.

### 2.4. EAP (in Mice)

EAP is first expressed during early embryogenesis (from the 2- to 8-cell stages to the blastocyst stage), but is not detectable in embryos older than 7.5 days post coitum (dpc) [37]. EAP is subsequently re-expressed in the thymus, intestine, and testis in adult mice [6]. The gene encoding EAP in mice is defined as Akp5 (Table 1) [1,17].

To examine the biological functions of EAP, Narisawa et al. [25] generated Akp5 null-mutated mice via homologous recombination, and the resulting mice had no obvious phenotypic abnormalities, indicating the nonessential role of EAP during embryonic development (Table 2). Dehghani et al. [26] independently created *Akp5* null-mutated mice called *EAP.ko* mice. *EAP.ko* preimplantation embryos had slower development and higher rates of degeneration in vitro, leading to fewer blastocysts. In vivo, *EAP.ko* mice had delayed parturition and reduced litter size. Furthermore, there was no compensation for the absence of *Akp5* in the embryos by other isozymes of ALP (Table 2). Overall, the study’s findings showed that the presence of an active *Akp5* is beneficial for mouse preimplantation development.

### 2.5. TNSALP

TNSALP is a heat-labile isozyme [4,13], which is expressed in the ICM of blastocysts and in migrating PGCs [28,37]. Additionally, TNSALP is expressed in developing neural tubes of mouse embryos between 8.5 dpc and 13.5 dpc [40]; however, it is expressed in skeletal tissues at later stages of embryogenesis. In adult mice, TNSALP is expressed in a wide variety of cell types, such as osteoblasts, neutrophils, renal tubules, capillaries in the brain, and myoid cells in the testis [25].

The gene encoding TNSALP is defined as *ALPL* in humans and as *Akp2* in mice (Table 1) [1,17]. ALPL consists of 12 exons, with the coding sequence beginning within the second exon [40,41,42]. Eleven exons are translated to form 507-amino-acid residues comprising TNSALP [40,41,42]. Exons 2–12 are contained within 25 kb of the DNA. *ALPL* has two promoters and two corresponding 5′ noncoding exons 1a and 1b, resulting in two different mRNAs. Transcription from the upstream promoter (1a) is used in osteoblasts, whereas the downstream promoter (1b) is used in the liver and kidney [43]. That *ALPL* has a dual-function promoter is also shown in humans [44], rats [45,46], and mice [47].

As already described, TNSALP is a zinc-containing metalloenzyme and functions as a dimeric molecule [16]. Based on the analysis of the *ALPL* cDNA, this gene encodes a preproprotein that is proteolytically cleaved to yield a signal peptide (comprising 17 amino acids) and a proprotein that is subsequently processed to generate the active mature peptide. TNSALP has five potential *N*-linked glycosylation sites that are essential for full activity [17]. The deduced active site of TNSALP is encoded by six exons comprising 15 amino acid residues and shares the same nucleotide sequence with other ALPs. There is a C-terminal hydrophobic domain that is replaced by a glycosylphosphatidylinositol (GPI) anchor, which is involved in inserting protein into the lipid bilayer of cells and liposomes [17].

Regarding the biological role of TNSALP, Kotobuki et al. [48] suggested an important role of TNSALP in osteoblastic function. They introduced small interfering RNAs (siRNAs) of the osteogenic-related genes (including runt-related transcription factor 2 (*RUNX2*), collagen type I α1 chain (*COL1A1*), *ALPL,* and osteocalcin (*OCN*)) into human ALP high-expressing osteoblast-like cells. They found that only *ALPL* siRNA inhibited matrix mineralization. In this instance, the expression of not only *ALPL* but also *COL1A1* and *RUNX2* were influenced by the inhibition of *ALPL*. In contrast, *OCN* expression was not affected by the inhibition of *ALPL*. Kotobuki et al. [48] concluded that TNSALP is a strong candidate for promoting matrix mineralization. Furthermore, as mentioned below, TNSALP promotes bone mineralization mainly by hydrolyzing extracellular inorganic PPi, which is a strong mineralization inhibitor.

#### 2.5.1. Creation of Akp2 KI or KO Mice

To examine the biological functions of TNSALP, Soriano and Millán’s groups generated *Akp2* KO mice using traditional gene targeting methods, and MacGregor et al. [27] generated KI mice (called *Alpl^tm1Sor^*). In these mice, the genomic *Akp2* sequence spanning from the middle of exon 2 to the middle of exon 6 was replaced with a *βgeo* cassette (an expression unit for both *lacZ* and neomycin resistance gene (*neo*^r^)) to enable the expression of β-gal under the control of the endogenous *TNSALP* promoter. Assessment of β-gal activity in the heterozygous progeny of *Alpl^tm1Sor^* showed that TNSALP was not expressed in PGC progenitors prior to gastrulation. The ß-gal activity was first found in an extraembryonic lineage destined to form the chorion. In homozygous null mutants, PGCs appeared unaffected, indicating that TNSALP is not essential for their development or migration. In contrast, *Alpl^tm1Sor^* mice had elevated plasma PLP levels and died from seizures caused by PL deficiency in cells of the central nervous system (CNS), resulting from a decrease in the hydrolysis of PLP to pyridoxal (PL) (Table 2) [31]. Additionally, Waymire et al. [28] reported that although *Alpl^tm1Sor^* mice had normal skeletal development, they exhibited elevated PLP levels, resulting in death approximately two weeks after birth (Table 2). However, the seizure phenotype can be rescued by the administration of PL and a semi-solid diet, but the rescued animals subsequently manifested dental dysplasia.

Narisawa et al. [25] generated *Akp2* KO mice (called *Alpl^tm1Jlm^* or *Akp2^−/−^*) by inserting a *neo^r^* cassette into exon 6 of the *Akp2* gene. The *Akp2* KO mice exhibited impaired growth, vitamin-B6-dependent seizures, impaired bone mineralization, and apnea, and died before weaning (Table 2) [25]. The plasma of the KO mice contained low ALP activity (Table 2) [22], whereas *Akp2* heterozygous mice had approximately 50% of the plasma ALP activity of WT mice and were healthy and fertile [25]. Narisawa et al. [25] speculated that TNSALP appears not to be essential for the initial events leading to bone mineral deposition but might play a role in maintaining the bone mineralization process after birth.

Notably, *Akp2* KO lines, *Alpl^tm1Sor^* and *Alpl^tm1Jlm^*, were compared in detail by Fedde et al. [30], who reported an increase in putative natural substrates (phosphoethanolamine, PPi, and PLP) for TNSALP in both lines. Bone abnormality was first observed around 10 days after birth, and osteopenia and fractures worsened at later stages, similar to the characteristics of infantile HPP.

#### 2.5.2. Creation of Conditional Akp2 KO Mice

Narisawa [33] generated a floxed mouse line *Alpl^flox/flox^*, by KI of an around 12 kb genomic sequence of *Akp2* in which two *loxP* sites (located in introns 2 and 4, respectively) and a cassette containing *neo*^r^ expression unit were transfected into the endogenous *Akp2* locus (Table 2). The results show that the floxed mouse was normal in the absence of Cre expression. However, in the presence of Cre, deletion of exons 3 and 4 occurred, which resulted in the inhibition of endogenous TNSALP expression. Narisawa [33] generated a Cre Tg mouse line, called *Col1a1-Cre*, in which bone-specific expression of Cre was driven under the control of *Col1a1* promoter. There was around 30% reduction in the plasma ALP levels in the bigenic offspring of *Alpl^flox/flox^* and *Col1a1-Cre* compared with that in the WT mice. The *Alpl^flox/flox^* line is thus useful for examining the function of TNSALP in any cell or tissue if appropriate Cre Tg lines are available.

Notably, Lomeli et al. [32] generated a Cre Tg mouse line, called *Alpl^tm1(cre)Nagy^*, by inserting *Cre* into the endogenous *Akp2* locus (Table 2), indicating that Cre expression is controlled by the *Akp2* promoter. When the *Alpl^tm1(cre)Nagy^* mice were crossed with the double-reporter line, Z/AP [49], carrying a floxed sequence containing *loxP* site, *lacZ* gene, *neo*^r^, transcription stopper, *loxP* site, and a gene encoding human alkaline phosphatase (hAP), the resulting bigenic offspring exhibited expression of reporter genes in PGCs at E9.5–10.5. At mid-gestational stages, it was expressed in the labyrinthine region of the placenta, the intestine, and the neural tube. Crossing *Alpl^flox/flox^* with *Alpl^tm1(cre)Nagy^* would result in the tissue-specific (i.e., PGCs, labyrinthine region of the placenta, and embryonic intestines and neural tubes) inhibition of TNSALP expression.

#### 2.5.3. Creation of Akp2 Mutant Mice through N-Ethyl-N-Nitrosourea (ENU) Mutagenesis

ENU mutagenesis is the forward genetics or phenotype-driven approach (phenotype to the gene) involving the screening of mice for mutant phenotypes without previous knowledge of the genetic basis of the mutation [50]. This approach is used when there is a need for highly efficient induction of point mutations in mice [51]. To perform ENU mutagenesis, male mice (aged 6- to 8-week old) are usually intraperitoneally injected with a solution containing ENU, an alkylating agent, which acts as a powerful mutagen [51].

In 2007, the Gena 328 mouse (called *Alpl^Hpp^*) exhibited low plasma ALP activity and late-onset skeletal abnormalities but a normal life span and no epilepsy [52]. A point mutation at the splice site for exon 8 of *Akp2* produced a truncated, inactive *TNSALP* having 276 residues rather than the 525 residues of WT *TNSALP.*

In 2009, the mouse mutant (called *Alpl**^ALPLD1^***) exhibited low plasma ALP activity [53]. An A to G point mutation in exon 5 of *Akp2* caused an Asp to Gly change at residue 109 of the TNSALP protein.

In 2012, the same group reported new mouse mutants, called *Alpl^BAP023^*, *Alpl^BAP026^,* and *Alpl^BAP027^*, produced by ENU mutagenesis [54]. *Alpl^BAP023^* carries a missense T to G mutation in exon 7 at nucleotide (nt) 755 (Leu to Pro at residue 251). *Alpl^BAP026^* has a splice site mutation in intron 9. *Alpl^BAP027^* carries a T to A mutation in exon 10 at nt 1194 (Ile to Asn at residue 395).

In 2015, a murine model for odontohypophosphatasia (odonto-HPP) was first generated by Foster et al. [55]. Odonto-HPP is a mild form of HPP characterized only by oral manifestations, including premature exfoliation of deciduous teeth. The odonto-HPP mouse model is characterized by a missense mutation that changes codon 116 from Ala to Thr, which has been identified similarly with autosomal dominant (AD) odonto-HPP. Overall, the odonto-HPP mouse model has around 50% WT plasma ALP activity of WT mice, with similar litter size, survival, and body weight.

#### 2.5.4. Creation of Tg Mice Overexpressing TNSALP

Furthermore, studies have examined the effect of the overexpression of TNSALP in various tissues on their behavior and function. Narisawa et al. [34] generated a Tg mouse line (called “*Col1a1-Tnap*”) expressing human *ALPL* under the control of an osteoblast-specific *Col1a1* promoter. The offspring of this line was healthy but exhibited increased mineralization of vertebrae bones (Table 2). Additionally, there was higher mineralization of osteoblasts from the Tg mice compared with that from non-Tg controls.

Sheen et al. [35] demonstrated that Tg mice carrying human *ALPL* and overexpressing TNSALP in vascular smooth muscle cells under the smooth muscle cell-specific transgelin (*Tagln*) promoter developed severe arterial medial calcification and reduced viability (Table 2).

Savinov et al. [36] determined the effect of TNSALP overexpression on vascular calcification and cardiovascular function using the endothelial-specific tunica intima endothelial kinase 2 (*Tie2*) promoter in mice. Mice with endothelial TNSALP overexpression, called “endothelial *TNSALP* mice,” survived well into adulthood and displayed generalized arterial calcification (Table 2). Genes associated with osteochondrogenesis (*Runx2*, bone γ-carboxyglutamate protein 2 (*Bglap2*), osteopontin (*Opn*), osteoprotegerin (*Opg*), and collagen type II α1 chain (*Col2a1*)) were upregulated in the aortas of the Tg offspring. At 23 weeks of age, endothelial *TNSALP* mice developed elevated blood pressure and compensatory left ventricular hypertrophy with normal ejection fraction. Savinov et al. [36] concluded that TNSALP has osteogenic potential in endothelial cells, promoting vascular calcification.

## 3. Various Biological Roles of TNSALP

As mentioned above, TNSALP is expressed in a variety of soft tissues, which do not undergo mineralization (e.g., kidney and liver), thereby casting some doubt on previous thought that TNSALP is involved only in osteoblastic function related to mineralization.

The various roles of TNSALP are discussed below.

### 3.1. Osteogenic System

**Figure 1:**
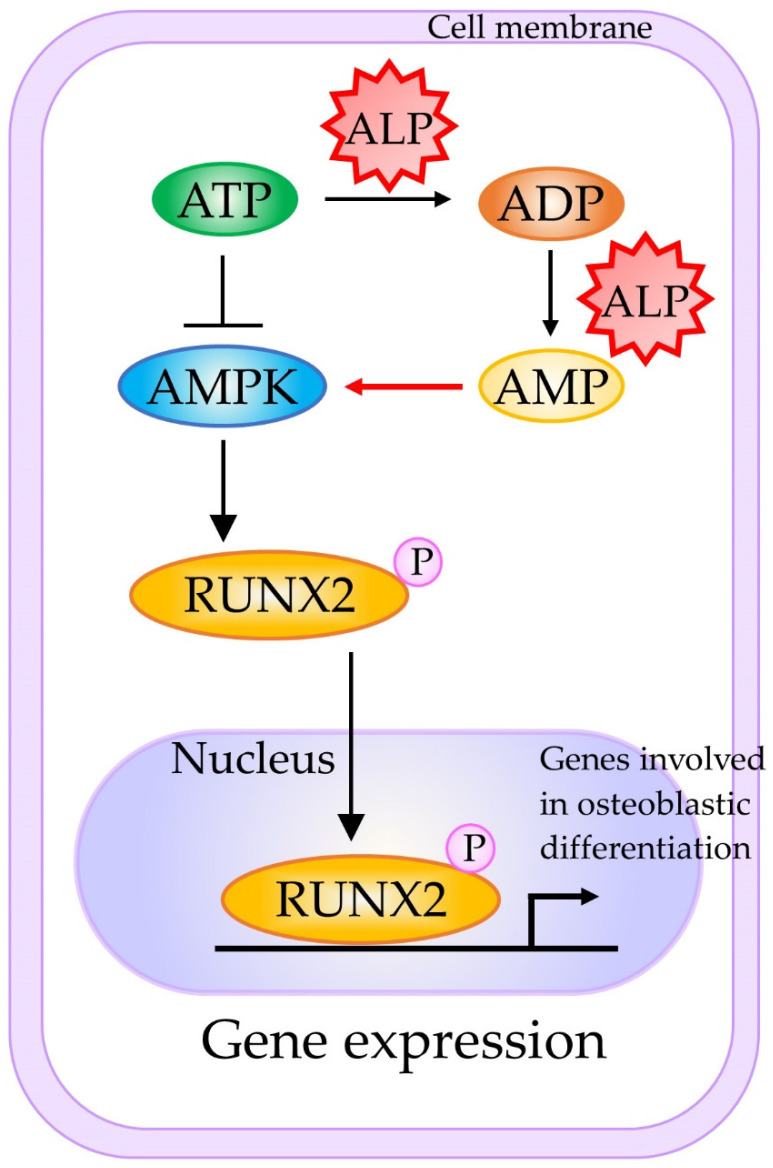
A schematic representation of the molecular mechanism underlying TNSALP-mediated activation of osteogenesis. Active AMPK and RUNX2 phosphorylation are preferentially associated with osteogenesis. Abbreviations: AMP, adenosine monophosphate; ADP, adenosine diphosphate; AMPK, AMP-activated protein kinase; ATP, adenosine triphosphate; MSCs, mesenchymal stem cells; RUNX2, RUNX family transcription factor 2. This figure was drawn in-house, based on the data shown in the paper of Chava et al. [57].

Mesenchymal stem cells (MSCs) are precursors of bone-producing osteoblasts, and the protein encoded by ALPL is enriched in the stem cell membrane, with involvement in ATP metabolism during cell differentiation. In Figure 1, the mechanism underlying the involvement of TNSALP in osteogenesis is shown. TNSALP in MSCs is tightly associated with AMP-activated protein kinase (AMPK) activation during osteogenesis [56,57]. In this system, AMPK is a key regulator to maintain cellular energy homeostasis. For example, Liu et al. [56] suggested that TNSALP deficiency in MSCs enhances ATP release and reduces ATP hydrolysis. The excessive extracellular ATP is, in turn, internalized by MSCs and causes an elevation in the intracellular ATP level, which consequently inactivates the AMPK pathway. However, AMPK is activated by the increased AMP, which is generated through the sequential degradation/hydrolysis reactions of polyP catalyzed by TNSALP. RUNX2 is a substrate of AMPK, which directly phosphorylates serine 118 in the DNA-binding domain of RUNX2 [57]. The resulting phosphorylated RUNX2 may activate bone matrix protein genes, leading to enhanced osteogenesis.

### 3.2. Lipid and Energy Metabolism of Fat Cells

According to Hernández-Mosqueira et al. [58], TNSALP is expressed in adipose tissue and in 3T3-F442A adipocytes. Moreover, there was an upregulation of glycerophosphate dehydrogenase, adiponectin (a recently described adipokine), and fatty acid-binding protein 4 (FABP4 levels; which is also called adipocyte protein 2 (aP2)) genes, but decreased expression of leptin in TNSALP knockdown cells. The latter three proteins are important in adipocyte systemic signaling and insulin sensitivity. Furthermore, inhibition of ALP activity in adipocytes by levamisole (a reversible inhibitor for TNSALP) reduced lipolysis and the expression of various lipogenic genes. Hernández-Mosqueira et al. [58] suggest that the activity of TNSALP might have a critical role in the energy balance of the adipocyte, probably participating in obesity and metabolic syndrome.

### 3.3. Neuronal System

Hanics et al. [31] assessed the function of TNSALP in a neuronal system in vivo using *Akp2* KO mice (*Akp2^−/−^*) and reported that TNSALP is expressed in the synaptic cleft and the node of Ranvier in normal adults. Ablation of TNSALP function resulted in a significant decrease in the white matter of the spinal cord accompanied by cellular degradation around the paranodal regions and a decreased ratio and diameter of the myelinated axons (Table 2). Hanics et al. [31] concluded that loss of TNSALP causes myelin abnormalities and synaptic dysfunction.

As previously shown, NSCs express TNSALP more abundantly. To gain insight into the functional role of TNSALP, Kermer et al. [59] applied a knockdown protocol using short hairpin RNA (shRNA) and retroviral infection toward cultured NSCs. TNSALP knockdown reduced the proliferation and differentiation of NSCs into neurons or oligodendrocytes, suggesting an important role of TNSALP in NSC proliferation and differentiation. Interestingly, the RNAi-mediated effect was abrogated by adding ALP (derived from a commercially available frozen product) to the culture medium, suggesting that TNSALP may act on the cell membrane. Kermer et al. [59] provided some reasons for this: (1) TNSALP may be involved in the metabolism of extracellular nucleotides through coupling with purine receptor function, (2) TNSALP may function as an ecto-phosphoprotein phosphatase with phosphodiesterase activity or mediate the hydrolysis of PPi to Pi, or (3) TNSALP may interact with extracellular matrix proteins, such as collagen, through a loop region of TNSALP.

Graser et al. [60] reported an increase in the expression of neuronal marker genes, such as RNA binding protein, fox-1 homolog (*C. elegans*) 3 (NEUN), contactin-associated protein-like 2 (CNTNAP2), neurexin 1 (NRXN1), doublecortin (DCX), and protein kinase C, alpha (PRKCA), as well as microtubule-binding proteins such as microtubule-associated protein 2 (MAP2) and microtubule-associated protein tau (TAU) during neurogenic differentiation in human SH-SY5Y neuroblastoma cell line overexpressing TNSALP. Graser et al. [60] concluded that TNSALP is capable of modulating intercellular communication in the CNS.

### 3.4. Immune System

TNSALP is known to be highly expressed in murine B lymphocytes when they are activated by either polyclonal mitogens or T helper cells (Th cells) [61]. Marquez et al. [62] extended this notion further and demonstrated that TNSALP expression is correlated with B cell differentiation into Ig-secreting cells. Similar results were obtained by another group [63], who found a parallel increase in electrophoretic mobility and TNSALP expression during B cell progression from the proliferative to the immunoglobulin (Ig) secretion stage. According to Marquez et al. [62], the phosphorylation–dephosphorylation mechanism may play a role in controlling the growth/differentiation rate in the B cell lineage.

Two ALP isoforms, IALP and TNSALP, are co-expressed in the mouse colon, with the latter predominating in colitis. Hernández-Chirlaque et al. [29] examined the role of TNSALP in T lymphocytes, using heterozygous *Akp2* KO mice called *B6.129S7-Akp2^tm1Sor^/J* (as homozygous *Akp2* KO mice are non-viable). In vitro primary cultures from these mice demonstrated that stimulated splenocytes and T lymphocytes showed decreased cytokine production and expression compared with WT cells. Decreased T cell activation was reproduced by levamisole in WT cells. Hernández-Chirlaque et al. [29] concluded that TNSALP modulates T lymphocyte function (specifically T-cell-dependent colitis).

### 3.5. Vascular Calcification

Ectopic vascular calcification (VC) is associated with aging, atherosclerosis, diabetes, and/or chronic kidney disease [64]. As mentioned previously, endothelium-specific expression of TNSALP in Tg mice resulted in VC [35,36]. Savinov et al. [36] suggested that TNSALP possesses osteogenic potential in endothelial cells, thus promoting VC.

Notably, Goettsch et al. [65] recently suggested that TNSALP can be a therapeutic target for cardiovascular calcification (CVC), which is associated with increased morbidity and mortality. According to them, CVC develops in several diseases and locations, such as in the tunica intima in atherosclerosis plaques, in the tunica media in type 2 diabetes and chronic kidney disease, and in aortic valves. At present, no treatment is yet available. Moreover, most CVC occurs in a mode similar to skeletal/dental mineralization caused by the overexpression of TNSALP. Thus, tools are now being developed to inhibit TNSALP activity using animal models of CVC. For instance, Andleeb et al. [66] discovered small drug-like molecules (a series of novel triazolyl pyrazole derivatives (10a-y)) as potent and selective inhibitors of human TNSALP. If these drugs are proven useful using animal models, they may be candidate compounds to target VC. Furthermore, Millan’s group [67] found a potent and orally bioavailable TNSALP inhibitor, called SBI-425, to inhibit pathological soft-tissue calcification in vivo.

### 3.6. Role of TNSALP in Renal Cell Carcinoma (RCC)

According to Sharma et al. [68], decreased activity of TNSALP is remarkable in renal cell carcinoma (RCC). Forced expression of *ALPL* cDNA in renal cell carcinoma (RCC) cell lines resulted in decreased migratory property and cell viability compared with the controls. Furthermore, the transfected cells exhibited increased apoptosis, suggesting the role of TNSALP in cell viability and apoptosis during renal tumorigenesis.

### 3.7. Role of TNSALP Fibroblastic-Like Cell Lines

Hui et al. [69] examined the effects of TNSALP on various aspects of cellular activity by transferring the *ALPL* into three fibroblastic-like cell lines: Chinese Hamster ovary (CHO), R1610, and Rat-2. The expression of TNSALP under the control of simian virus 40 (SV40) promoter (but not R1610) caused a decrease in the proliferation and migration of CHO and Rat-2 cells, indicating the cell proliferation and migration inhibitory effect of TNSALP.

### 3.8. Role of TNSALP in the Hair-Inductive Capacity of Human Dermal Papilla Spheres

Human dermal papilla (DP) regulates the overlying epithelial cells and plays a key role in regulating hair growth and regeneration. To examine the effect of TNSALP in human DP cells, Kwack et al. [70] overexpressed TNSALP in DP spheres and carried out a hair reconstitution assay. Overexpression of TNSALP significantly increased hair follicle induction, which was closely associated with the Wnt/β-catenin signaling pathway. Additionally, there was a significant increase in the expression levels of Wnt/β-catenin target genes, such as axis inhibition protein 2 (AXIN2), *versican* (VCAN), and lymphoid enhancer-binding factor 1 (LEF1), in DP cells overexpressing TNSALP (Table 3). Moreover, overexpression of TNSALP significantly affected the expression level of bone morphogenetic protein 4 (BMP4) but did not affect the expression profiles of bone morphogenetic protein 2 (BMP2), *noggin* (NOG), and fibroblast growth factor 7 (FGF7) (Table 3). Based on these findings, it was concluded that TNSALP augments the hair-inductive capacity of human DP spheres by regulating the Wnt/β-catenin signaling pathway and possibly by maintaining the characteristics of the DP.

### 3.9. TNSALP May Be Involved in Premature Bone Aging

As mentioned previously, Liu et al. [56] determined the mechanism of bone aging and found that TNSALP deficiency in stem cells enhanced the release of ATP and reduced its hydrolysis to cause extracellular ATP boost. When internalized by MSCs, ATP inactivated the AMPK α subunit (AMPKα) cell signaling pathway (master regulator of cellular energy homeostasis), contributing to MSC cell fate switch by impairing their ability to grow and proliferate. The work was conducted in vitro and in a mutant mouse model (*Alpl^+/-^*) exhibiting premature aging, followed by metformin treatment to improve the function of endogenous MSCs by reactivating the cell signaling pathway. Liu et al. [56] demonstrated that knockdown of *Akp2* induced premature bone aging, which was characterized by bone mass loss and marrow fat gain. Liu et al. [56] then reactivated the pathway using the diabetes drug metformin to restore the ability of stem cells to grow and differentiate into bone-producing osteoblasts and prevent bone aging.

### 3.10. Mitochondrial TNSALP Controls Thermogenesis

Adaptive thermogenesis has been defined as the change in energy expenditure following acute and/or long-term overfeeding and underfeeding. Recent data have indicated that thermogenic fat cells use creatine to stimulate futile substrate cycling, dissipating chemical energy as heat based on the super-stoichiometric relationship between the amount of creatine added to mitochondria and the quantity of oxygen consumed. Sun et al. [72] recently provided direct evidence for the critical role of TNSALP as a phosphocreatine phosphatase in the futile creatine cycle using mice. The thermogenic fat cells have a high degree of phosphocreatine phosphatase activity, caused by the action of mitochondrial TNSALP capable of hydrolyzing phosphocreatine to initiate a futile cycle of creatine dephosphorylation and phosphorylation. The expression of mitochondrial TNSALP is reportedly triggered in mice exposed to cold conditions, and its inhibition in isolated mitochondria leads to a loss of futile creatine cycling. Genetic ablation of TNSALP in adipocytes reduces whole-body energy expenditure, leading to rapid-onset obesity in mice.

## 4. Possible Involvement of TNSALP in Differentiation and Maintenance in Juvenile State

Although there is a strong correlation between TNSALP expression and undifferentiated juvenile cells, the role of TNSALP in the induction, maintenance, and differentiation of these cells remains unknown. For example, TNSALP expression is induced during the reprogramming of somatic cells to iPSCs [73]. TNSALP expression is rapidly reduced when juvenile cells, such as ESCs or iPSCs, are induced to differentiate [74]. Based on these findings, we hypothesized that TNSALP expression might cause changes in cellular behavior, which may be associated with cellular differentiation or di-differentiation. Therefore, the role of TNSALP in cell differentiation is discussed in detail below.

### 4.1. TNSALP as an Early Marker Expressed in Intermediate Cells towards iPSCs

**Figure 2:**
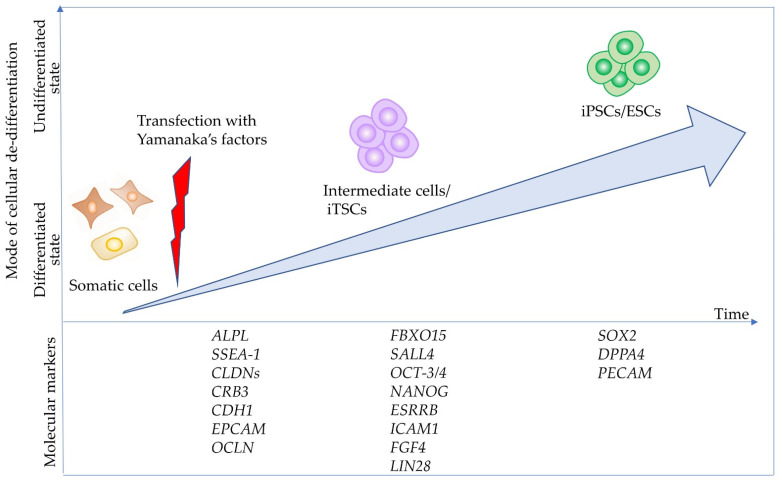
Cell state and molecular events during the reprogramming of somatic cells into induced pluripotent cells (iPSCs). When complete reprogramming occurs, somatic cells are successfully converted into iPSCs. The resulting iPSCs can be further reprogrammed into naïve iPSCs through transfection with vectors carrying Yamanaka’s factors or via treatment with chemicals. Additionally, somatic cells are converted into “intermediate cells” called iTSCs, when partial reprogramming occurs. There are at least three phases with respect to (de-)differentiation (“somatic state” which may correspond to the initiation stage, “intermediate state” which may correspond to the maturation stage, and “pluripotent state” which may correspond to the stabilization stage), according to the paper of Samavarchi-Tehrani et al. [76]. Importantly, several molecular markers define each of the above phases, as per Samavarchi-Tehrani et al. and Polo et al. [76,77]. This figure was drawn in-house, based on the data shown in the paper of Adachi et al. [78]. Abbreviations: ALPL, gene encoding tissue-nonspecific alkaline phosphatase (TNSALP or TNAP); CDH1, E-cadherin; CLDNs, claudins; CRB3, crumbs cell polarity complex component 3; EPCAM, epithelial cell adhesion molecule; ESRRB, estrogen-related receptor Beta; FBXO15, F-box protein 15; ESCs, embryonic stem cells; FGF4, fibroblast growth factor 4; DPPA4, developmental pluripotency associated 4; ICAM1, intercellular adhesion molecule 1; LIN28, Lin-28 homolog; NANOG, Nanog homeobox; OCLN, occludin; OCT-3/4, octamer-binding transcription factor-3/4; PECAM, platelet endothelial cell adhesion molecule; SALL4, spalt-like transcription factor 4; SOX2, sex-determining region Y-box 2; SSEA-1, stage-specific embryonic antigen 1.

During reprogramming, candidate iPSC colonies generate approximately 20 days after transfection with reprogramming factors and are generally examined for the expression of pluripotent markers, such as octamer-binding transcription factor-3/4 (*OCT-3/4*), sex-determining region Y-box 2 (*SOX2*), stage-specific embryonic antigen 4 (SSEA-4), tumor rejection antigens (TRA)-1-60 (TRA-1-60), and TRA-1-80, using molecular methods [73]. Moreover, ALP expression is discernible in the colonies as early as 14 days post-transduction and can be used as a marker for the early identification of iPSCs. Brambrink et al. [73] examined the timing of known pluripotency marker activation during mouse iPS cell derivation and observed that ALP was activated first, followed by stage-specific embryonic antigen 1 (SSEA-1). Expression of the homeobox-containing transcription factor Nanog, whose name stems from the Celtic/Irish mythical word Tír na nÓg, and the endogenous *OCT-3/4* gene, marking fully reprogrammed cells, were only observed late in the process. According to David and Polode [75], ALP-positive cells are considered as “intermediate cells” being reprogrammed into undifferentiated cells, such as iPSCs (Figure 2).

**Figure 3:**
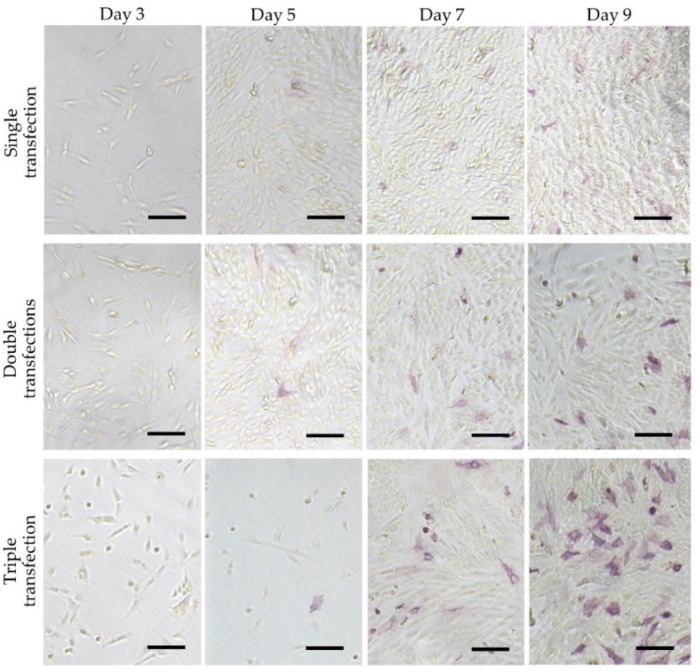
Cytochemical evaluation of ALP activity in HDDPCs after repeated transfections with the reprogramming factors. HDDPCs (P05 line) were transfected with Yamanaka’s four reprogramming factors once, twice, or three times. The treated cells were subjected to cytochemical staining for ALP activity at 3, 5, 7, and 9 days after the final transfection. These photographs were originally constructed using data used in the paper of Soda et al. [80]. Bar  =  500 μm.

Generally, stem cells included in the adult organs/tissues (which are called “somatic stem cells” or “adult stem cells”) express certain stemness markers (such as Oct-3/4 and ALP). These stem cells are more easily reprogrammed than the other somatic cells because adult neural stem cells (which show endogenous high expression of pluripotent genes, such as Sox2 and c-Myc) are known to be reprogrammed with only two factors [79]. These suggest that ALP-positive somatic cells may be easily reprogrammed to iPSCs by reprogramming factors other than ALP-negative cells. To confirm this point, Inada et al. [80] examined the potential of five isolated primary human deciduous tooth-derived dental pulp cells (HDDPCs) to induce iPSCs after reprogramming. They demonstrated that two lines highly enriched with ALP-positive cells were successfully reprogrammed. However, three ALP-negative lines failed to be reprogrammed. Notably, HDDPCs with a high degree of ALP activity (also associated with increased expression of ALPL mRNA) exhibited an active proliferation rate and expression of stemness factors, such as OCT-3/4, SOX2, and NANOG. In contrast, HDDPCs with a low degree of ALP activity did not show such properties. Based on these findings, Inada et al. [80] speculated that ALP-positive cells included in HDDPCs may be stem cells or intermediate cells between fully differentiated cells and undifferentiated cells such as iPSCs. Similarly, Soda et al. [81] reported that only one cell line exhibiting ALP activity among six cell lines was successfully reprogrammed into iPSCs by reprogramming factors, with the other five lines remaining negative for ALP activity after 10 days of transfection. However, when the five lines were transfected again with the reprogramming factors, four exhibited ALP activity approximately 10 days after the second transfection (shown in Figure 3), and were successfully reprogrammed to form iPSCs. Based on these findings, it can be concluded that the appearance of intermediates cells that express TNSALP is necessary to form iPSCs. Notably, according to Štefková et al. [82], little is known about the inability to isolate ESCs from *Akp2* KO embryos.

### 4.2. Possible Involvement of Wnt/β-Catenin Signaling Pathway in Generation of iPSCs

As previously mentioned, the successful development of somatic cells (potentially ALP-negative like fibroblastic cells) to iPSCs is always associated with the appearance of ALP-positive cells (“intermediate cells”). However, it is necessary to examine the mechanism responsible for changing ALP-negative cells to ALP-positive cells after transfection with reprogramming factors. It has been speculated that several signaling pathways may involve in this process, among which the Wnt/β-catenin signaling has been examined.

**Figure 4:**
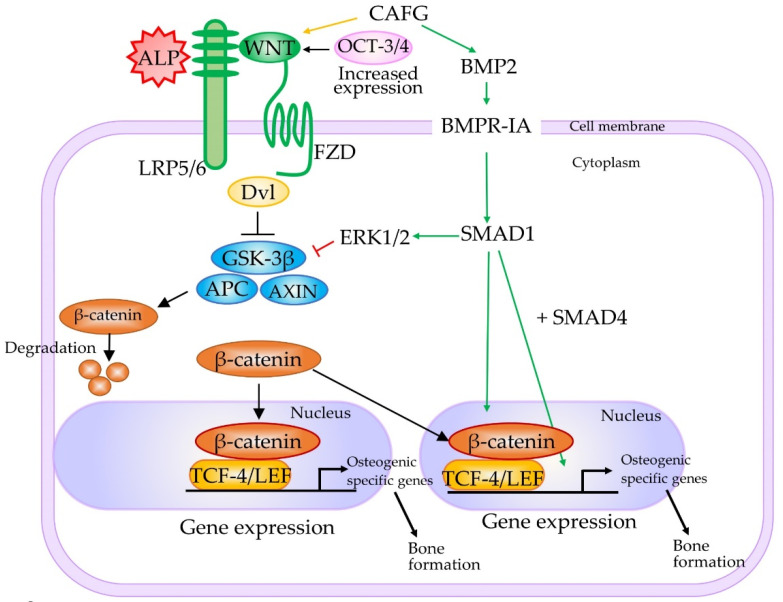
Molecular mechanisms underlying TNSALP-mediated bone marrow stem cells (BMMSC) lineage switching. According to Liu et al. [86], overexpressed TNSALP interacts with low-density lipoprotein-related receptors 5 and 6 (LRP5/6) molecules, one of the important elements of the canonical Wnt/β-catenin pathway, to inhibit phosphorylation of glycogen synthase kinase-3β (GSK-3β). As a result, the nuclear location of β-catenin is accelerated, leading to activation of downstream genes that are involved in osteogenesis and controlled by T cell factor-4 (TCF-4)/lymphoid enhancer factor (LEF) (TCF-4/LEF) proteins. These osteogenesis-related downstream genes may also be regulated by the BMP2-related signaling pathway. β-Catenin can also interact with pluripotency-related genes, such as Krüppel-like factor 4 (KLF4), octamer-binding transcription factor-3/4 (OCT-3/4), and sex-determining region Y-box 2 (SOX2). Abbreviations: ALP, alkaline phosphatase; APC, adenomatous polyposis coli tumor suppressor; AXIN, axis inhibition protein; BMP2, bone morphogenetic protein 2; BMPR-IA, bone morphogenetic protein receptor type IA; CAFG, caviunin 7-O-[β-D-apiofuranosyl-(1-6)-β-D-glucopyranoside]; Dvl, dishevelled (Dsh) protein; ERK1/2, extracellular signal-regulated kinase (ERK)1/2; FZD, Frizzled; SMAD 1, SMAD family member 1; SMAD4, SMAD family member 4. This figure was drawn in-house, based on the data shown in the paper of Liu et al. [86].

The Wnt family consists of a large number of secreted glycoproteins that are involved in multiple cellular events, such as cell proliferation, differentiation, and apoptosis through β-catenin-dependent canonical and β-catenin-independent noncanonical pathways [83,84]. In the canonical Wnt/β-catenin pathway, Wnt proteins bind to the seven transmembrane receptors, called Frizzled (FZD), and the low-density lipoprotein receptor-related protein (LRP5/6) co-receptors located on the cell surface. The binding of Wnt protein to its receptors leads to the phosphorylation of the disheveled (Dsh) protein (Dvl) through its association with Axin and the adenomatous polyposis coli tumor suppressor (APC). Additionally, this binding inhibits the phosphorylation of β-catenin by glycogen synthase kinase-3β (GSK-3β) and the cytosolic accumulation of β-catenin, resulting in the translocation of unphosphorylated β-catenin to the nucleus. In the nucleus, β-Catenin interacts with members of the T cell factor-4 (TCF-4)/lymphoid enhancer factor (LEF) family of transcription factors to activate the expression of downstream genes involved in proliferation (e.g., *MYC*, cyclin D1 (*CCND1*), peroxisome proliferator-activated receptor δ (*PPARD*)), stem cell fate (e.g., achaete-scute family bHLH transcription factor 2 (*ASCL2*)), survival (e.g., ATP binding cassette subfamily B member 1 (*ABCB1*), baculoviral IAP repeat-containing 5 (*BIRC5*) (also known as survivin)), differentiation (e.g., inhibitor of DNA-binding 2 (*ID2*), transcription factor 4 (*TCF4*; also known as *ITF2*), ectoderm-neural cortex protein 1 (*ENC1*)), migration (e.g., matrix metallopeptidase 7 (*MMP7*), matrix metallopeptidase 14 (*MMP14*)), and angiogenesis (e.g., vascular endothelial growth factor (*VEGF*)) [85]. The pathway is schematically shown in Figure 4.

The importance of the Wnt/β-catenin signaling pathway in somatic cell reprogramming was confirmed by Kimura et al. [87], who observed high expression of Wnt2 in the early stage of reprogramming. Furthermore, *Wnt2* knockdown suppressed the nuclear accumulation of β-catenin and reduced reprogramming efficiency, whereas *Wnt2* overexpression promoted nuclear accumulation of β-catenin and enhanced reprogramming efficiency. Moreover, experiments using drugs that regulate the Wnt pathway confirmed the importance of the nuclear accumulation of β-catenin in reprogramming. Overall, it can be concluded that the upregulation of *Wnt2* and subsequent accumulation of β-catenin in the nucleus are key events in reprogramming.

**Figure 5:**
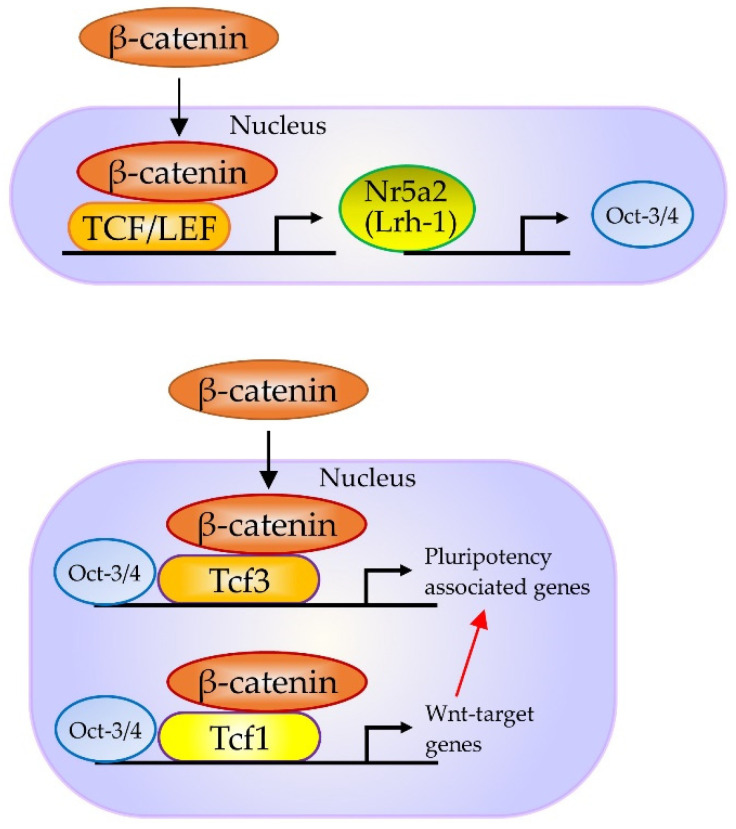
In murine embryonic stem (ES) cells, WNT/β-catenin/NR5A2 (LRH-1) is known to modify pluripotency genes expression. According to Tanaka et al. [91], WNT3A activates the WNT/β-catenin pathway and increases the expression of Nr5a2, which could directly enhance the expression of core pluripotency factors T-box transcription factor 3 (Tbx3), Nanog and Oct-3/4; however, the activation of this pathway is limited when the cells are treated with WNT3A alone. Abbreviations: NR5A2, nuclear receptor subfamily 5 group A member 2; LRH-1, liver receptor homolog-1. This figure was drawn in-house, based on the data shown in the paper of Tanaka et al. [91].

Notably, in mouse embryonic stem (ES) cells, stabilized β-catenin forms a complex with Oct-3/4 and enhances the activity of Oct-3/4, thus increasing pluripotency through a TCF-4/LEF-independent mechanism [88]. Furthermore, β-catenin and TCF3 target Nr5a2 (also known as liver receptor homolog-1 (Lrh-1)) and Nr5a2, in turn, directly activating the expression of Tbx3, Nanog, and Oct-3/4 in mouse ES cells (Figure 5) [89]. Moreover, Nr5a2 can replace Oct-3/4 in the reprogramming of mouse somatic cells [90]. According to Tanaka et al. [91], activation of β-catenin targets can maintain pluripotency and enhance cell reprogramming.

Furthermore, the relationship between the Wnt/β-catenin signaling pathway and upregulation of TNSALP expression has been examined. Si et al. [92] reported that Wnt3A (a representative canonical Wnt ligand) induced ALP activity in MSCs, which was inhibited by Dickkopf WNT signaling pathway inhibitor 1 (*Dkk1*) and dominant-negative *Tcf4*. Moreover, silencing *Wnt5a* expression enhanced the Wnt3a-mediated increase in *ALPL* expression in a dental follicle cell line [93]. Treatment with 6-bromoindirubin-3′-oxime (BIO), a drug capable of inhibiting GSK-3β phosphorylation and its activity, increased *ALPL* expression in cells, thereby activating the Wnt/β-catenin signaling pathway [94]. BIO enhanced *ALPL* mRNA expression in canine bone MSCs, indicating the importance of Wnt/β-catenin signaling pathway during somatic cells reprogramming to iPSCs.

### 4.3. Possible Involvement of BMP-2 Signaling Pathway in the Generation of iPSCs 

BMP-2 is one of the most potent bone-inducing agents in osteoblast differentiation. Moreover, it induces osteogenic trans-differentiation of fibrogenic, myogenic, and adipogenic cells both in vitro and in vivo [95]. A Wnt autocrine loop (a Wnt autocrine/paracrine loop) mediates the induction of *ALPL* and mineralization by BMP-2 in pre-osteoblastic cells [96,97]. Because LRP5/6 acts as a coreceptor for Wnt proteins, loss of function of LRP5/6 leads to osteoporosis (osteoporosis-pseudoglioma syndrome (OPPG)), and a specific point mutation in this same receptor results in high bone mass.

As shown in Figure 4, signaling pathways related to osteogenesis seemingly overlap with those related to iPSCs genesis. TCF-4/LEF proteins, to which activated β-catenin binds, activate downstream genes of the Wnt/β-catenin signaling pathway, including osteogenesis-related proteins, such as RUNX2, osterix (OSX), OPN, and TNSALP [98]. Additionally, BMP-2 stimulation activated RUNX2 and TNSALP expression, thus increasing the proliferation of osteoblastic cells. Zhang et al. [99] reported that the Wnt/β-catenin signaling pathway enhances iPSCs induction at the early stage of reprogramming through the interaction of β-catenin with Yamanaka’s reprogramming factors (Krüppel-like factor 4 (KLF4), OCT-3/4, and SOX2), further enhancing the expression of pluripotency circuitry genes. These findings suggest that Wnt signaling may be implicated in somatic cell reprogramming.

Notably, as already shown by Kimura et al. [87], transfection with shRNAs against *Wnt2* caused a reduction in the level of Wnt2, reducing the number of cells showing nuclear localization of β-catenin and reducing the number of ALP-positive or NANOG-positive colonies. These findings suggest that Wnt2 is required for both the nuclear localization of β-catenin and the initiation of reprogramming. Moreover, Wnt2 promotes tumor progression and is involved in epithelial–mesenchymal transition (EMT) events [100]. Mesenchymal–epithelial transition (MET) occurs at the initial step of fibroblast to iPS cell reprogramming. Prolonged activation of the Wnt2-mediated Wnt/β-catenin signaling pathway may inhibit MET in fibroblasts. Thus, for proper fibroblasts to iPS cell reprogramming, the Wnt2-mediated Wnt/β-catenin signaling pathway has to be “on” in the initial step of reprogramming, but “off” in the later steps.

Samavarchi-Tehrani et al. [76] reported that treatment with Yamanaka’s factors increased the expression of several epithelial cell-related genes during the “initiation phase” of reprogramming (first 5 days). Specifically, there was an increase in the expression of epithelial cell markers, such as E-cadherin (*Cdh1*), Claudins-2, -4, and -11 (*Cldns-3, -4, -7,* and *-11*), occludin (*Ocln*), epithelial cell adhesion molecule (*Epcam*), and Crumbs homolog (*Crb3*), a decrease in the expression of mesenchymal-specific genes, such as zinc finger protein SNAI1 (*Snail*), *Slug*, zinc finger E-box binding homeobox 1 (*Zeb1*), and zinc finger E-box binding homeobox 2 (*Zeb2*), and a loss of fibroblastic marker genes during the initiation of MET. siRNAs targeting *Cdh1* are known to inhibit the appearance of ALP-positive colonies, suggesting that MET is a key event that controls the success of cell reprogramming. To examine the role of the BMP signaling pathway in MET, knockdown of survival of motor and autonomic neurons 1 (*Sman1*) suppressed the formation of ALP-positive cells. Similarly, knockdown of SMAD family member 4 (*Smad4*), BMP type II receptor (*BMPRII*), or BMP type I receptor activin receptor-like kinase 3 (*Alk3*) suppressed the de-differentiation of ALP-positive cells. Based on these findings, MET and BMP signaling could be necessary at the initiation phase of reprogramming.

## 5. TNSALP as a Signal Regulator 

As previously mentioned, TNSALP is involved in a variety of cell behavior, including cellular proliferation, cell movement, and cell differentiation/di-differentiation. Evidence suggesting that TNSALP may function as a signal regulator is discussed in the preceding sections.

### 5.1. TNSALP May Be Involved in Lineage Switching

BMMSCs isolated from HPP patients exhibit decreased capacity to differentiate into osteocytes and increased capacity to differentiate into adipocytes [101]. Specifically, in vitro assay showed a decrease in osteogenic differentiation as evidenced by a decrease in the expression of RUNX2 and OCN, and an increase in adipogenic differentiation as evidenced by positive Oil Red staining and an increase in peroxisome proliferator-activated receptor gamma (PPARγ) expression [101]. Furthermore, in vivo transplantation assay showed a decrease in the differentiation of BMMSCs isolated from HPP patients into the osteogenic lineage, similar to results obtained in BMMSC isolated from *Akp2* KO mice.

Liu et al. [86] examined the regulatory role of TNSALP in BMMSC lineage switching and observed a decrease in the expression of Frizzled class receptor 2 (FZD2), Frizzled class receptor 9 (FZD9), and LRP5/6. Overexpression of TNSALP in BMMSC from HPP patients caused an increase in the level of LRP6 alone. Downregulation of TNSALP in normal BMMSC caused the partial inhibition of the WNT3A-mediated activation of the canonical Wnt/β-catenin pathway. The downregulation of LRP6 caused a similar phenomenon. Anti-TNSALP antibody successfully immunoprecipitated LRP5/6. Based on these findings, it was speculated that TNSALP does not interact with GSK-3β or β-catenin (*CTNNB1*) directly, but with LRP5/6 molecules to inhibit phosphorylation of GSK-3β, accelerate nuclear location of β-catenin, and activate genes controlled by TCF-4/LEF proteins. This process is shown in a schematic diagram in Figure 4. The findings of Liu et al. [86] appear to be the first to reveal the signal regulator role of TNSALP, which regulates the lineage switching of BMMSCs by regulating the LRP5/6/GSK-3β cascade. However, studies are yet to identify the site of TNSALP capable of interacting with LRP5/6.

Notably, Najar et al. [71] demonstrated that adipose tissue-derived MSCs contained two populations, so-called ALDH+ and ALDH-, based on the aldehyde dehydrogenase (ALDH) activity known to be a classical feature of stem cells. When transcriptome analysis of both cell populations was carried out, ALDH+ cells exhibited higher expression of osteogenic differentiation-related genes such as *RUNX2, OSX* and *OPG* than ALDH- cells. According to Najar et al. [71], the potential of differentiation towards the osteogenic lineages seems to be not equal and ALDH- cells present a more differentiated state than ALDH+ cells. In this context, it will be of interest to examine which types of adipose tissue-derived MSCs are related to TNSALP-mediated lineage switching. 

### 5.2. Overexpression or Suppression of ALPL May Affect the Expression of Some Genes

Overexpression of *ALPL* was associated with decreased expression of smooth muscle aortic alpha-actin (*ACTA2*) and *TAGLN* [35]. Furthermore, siRNA-mediated suppression of *ALPL* mRNA resulted in suppression of *COL1A1* and *RUNX2* expression [48]. Nakamura et al. [102] reported that treatment of murine osteoblast precursor cells with revamisol decreased the expression of osteogenesis-related proteins: *Runx2*, Sp7 transcription factor (*Sp7*), *Bglap2*, and dentin matrix protein 1 (*Dmp1*). In contrast, overexpression of TNSALP in osteoblasts isolated from *Akp2* KO mice caused increased expression of *Runx2*, *Bglap2*, and *Dmp1*. The genes affected by the level of *TNSALP* are listed in Table 3**.**

### 5.3. Expression of ALPL May Be Affected by Some Genes

The mechanism underlying the regulation of TNSALP remains unclear. Previous studies identified transcription factor binding motifs on the *ALPL* promoter, such as TATA box, Sp1 binding site, E-box-like sequences, and vitamin D response element-like motifs in humans [103] and mice [104]. Notably, Štefková et al. [81] performed in silico analysis and identified several binding sites for transcription factors associated with pluripotency, such as OCT-3/4, NANOG, transcription factor 3 (TCF3), and Forkhead box D3 (FOXD3) in the ALPL promoter.

To date, several transcription factors (differentiation-inducing factor-1 (DIF-1) [105], forkhead transcription factor FOXO1 (previously known as *FKHR*) [106], distal-less homeobox 5 (*DLX5*)-binding cis-acting element [107], and p107 retinoblastoma family transcription factor [108]) have been shown to bind the *ALPL* promoter. For example, Hatta et al. [109] examined the expression of FOXO1, a regulator of hepatic glucose metabolic and proapoptotic genes, in osteogenic cells and the effect of FOXO1 on transcription of the *ALPL* gene. RT-PCR and immunoblot analyses revealed the expression of FOXO1 in osteogenic cells, such as MC3T3-E1, SaOS2, and UMR 106. Consequently, it was demonstrated that overexpression of FOXO1 stimulated *ALPL* promoter activity through the forkhead response element in its promoter. These results suggest that *ALPL* is a target gene regulated by FOXO1 and that FOXO1 contributes to osteoblast maturation and osteogenesis. Yusa et al. [108], using hematopoietic cells, demonstrated that Sp3 transcription factor (Sp3) could bind to the fragment spanning around 150 bp upstream from the transcription initiation site of *ALPL*, suggesting that Sp3 activates the *ALPL* promoter in hematopoietic cells.

As previously described, TNSALP expression is stimulated by BMP-2 treatment. However, how BMP-2 induces TNSALP expression is not clearly understood. Kim et al. [107] attempted to determine the mechanism of BMP-2 on TNSALP using the murine *Akp2* promoter, which contains a *Dlx5*-binding cis-acting element. They demonstrated that Dlx5 transactivates *Akp2* expression directly by binding to the *Dlx5*-binding cis-acting element.

## 6. Therapeutic Aspect of TNSALP

Increase in serum ALP activity commonly originates from liver and bone. Consequently, the examination of serum ALP activity is of particular important in assessing possible hepatobiliary disease. The response of the liver to any form of biliary obstruction induces the synthesis of ALP by hepatocytes, which results in canalicular leakage of ALP into the hepatic sinusoid and subsequent inflow into blood vessels to increase serum ALP activity. A similar increase is seen in patients with advanced liver cancer or widespread secondary hepatic metastases [110]. The increased level of serum ALP has also been attributed to an increased activity of ALP, which is localized in the plasma membrane of osteoblasts before extracellular release, and also correlates with the extent of the bone diseases (i.e., Paget disease or rickets/osteomalasia). Notably, ALP is normally elevated in growing children and adults over the age of fifty [111].

As already described, serum ALP activity and increased amount of PPi are known to be associated with patients with HPP, an inheritable disease caused by mutations in the *ALPL* gene [112,113]. HPP has a broad-range of severity from stillbirth to pathological fractures in adulthood, depending on the degree of *ALPL* deficiency [112,113,114]. Patients with the most severe type of HPP experience respiratory failure soon after birth, thus requiring respiratory support [14]. To date, more than 350 mutations have been reported in the *ALPL* gene mutations database (www.sesep.uvsq.fr/03_hypo_mutations.php (accessed on 24 November 2021)).

Enzyme replacement therapy (ERT) using bone-targeting recombinant ALP Strensiq® (asfotase alfa) that is comprised by catalytic domain of TNSALP, Fc domain of human immunoglobulin, and 10 asparagine peptides is now available in several countries and has improved the prognosis of patients [115,116]. Although ERT is expensive, subcutaneous injections three or six times a week can improve the medical management of HPP patients [117,118].

As an alternative to ERT in HPP treatment, gene-engineered MSC-based transplantation therapies are considered [99]. For example, Nakano et al. [119] introduced a gene correction targeting vector into iPSCs isolated from two HPP patients by TALENS to correct c.1559delT mutation, the most frequent mutation in Japanese HPP patients. After selection with antibiotics, some clones with successful gene correction were obtained. These clones exhibited ALP activity. Osteoblasts differentiated from the corrected iPSCs exhibited high ALP activity and some calcification in vitro. Overall, the gene-corrected iPSCs can be used as a source for cell replacement therapy for HPP patients. On the other hand, viral gene delivery-based therapy is also considered as an alternative to ERT. For example, Yamamoto et al. [120] tested the possible use of a lentiviral vector (carrying human *ALPL* with deca-aspartate motif (D_10_) at the C-terminus (TNALP-D_10_), which is hereinafter referred to as TNALP-D_10_). They injected the vector into the jugular vein of 1-day-old *Akp2^−/−^* mice, a murine model for severe infantile HPP, to rescue the HPP phenotype. The injected mice exhibited no epilepsy and survived more than 10 months with an improved bone phenotype. Matsumoto et al. [121] attempted to use a recombinant adeno-associated virus (rAAV) because rAAV is thought to be safer than a lentiviral vector that potentially integrates their genome into host chromosomes. They performed an intravenous injection of rAAV expressing bone-targeted TNALP-D_10_ into *Akp2^−/−^* mice [25]. To develop a safer and more clinically applicable transduction strategy for HPP gene therapy, the same group [122] recently examined the efficacy of muscle-directed expression of TNALP-D10 using an rAAV with a serotype of 8 (which is hereinafter referred to as rAAV-8). Injection of this vector into the bilateral quadriceps of neonatal *Akp2^−/−^* mice resulted in healthy mice with more than 3 months of survival. Recently, Kinoshita et al. [123] further extended the work of Nakamura-Takahashi [122] by performing a single intramuscular administration of rAAV-8 encoding TNALP-D10 to increase the life span and improve the skeletal and dentoalveolar phenotypes in *Akp2^−/−^* mice within 5 days after birth. Treated mice exhibited elevated serum ALP activity, suppressed plasma PPi, extended life span, no sign of rickets, normal-like bone microstructure, and no ectopic calcifications in the kidneys, aorta, coronary arteries, or brain. They suggest that rAAV-mediated, muscle-specific expression of TNALP-D_10_ through intramuscular administration of therapeutic rAAV-8 may be a promising alternative to ERT to treat severe infantile forms of HPP.

As mentioned above, the most frequently used animal model for HPP is *Akp2^−/−^* mice. Williams et al. [124] recently generated another animal model, namely, a sheep model for HPP (“HPP sheep”), by introducing a single point mutation (1077 C  >  G) in *ALPL* using a CRISPR/Cas9 system. The results show that HPP sheep exhibited reduced serum ALP activity, decreased tail vertebral bone size, and metaphyseal flaring, consistent with the mineralization deficits observed in human HPP patients. Overall, the animal model would be beneficial for developing treatment strategies for HPP.

Molecular analysis of HPP patients revealed the presence of numerous mutations in the *ALPL* gene, as previously mentioned. To examine the function of these mutations in a short time, transfection of mutated *ALPL* into cultured cells, such a COS-1 cells, would be very convenient. For example, Takinami et al. [125] transfected F310L or F310L and V365I (F310L/V365I) in *ALPL* from HPP patients into COS-1 cells and observed a 67% and 31% reduction, respectively, in ALP activity compared with that in WT mice.

As previously mentioned, for treating HPP patients, administration of drugs, such as Strensiq® (asfotase alfa), is now being frequently used as ERT. However, due to drug dependence and the Quality of Life (QOL) problem, an alternative to ERT has been explored. In this context, cell-based transplantation of gene-corrected cells (i.e., gene-corrected BMMSCs derived from HPP patients) or local (muscle-targeted) administration of therapeutic rAAV would be the best option. Particularly, in the former case, gene correction of the mutated *ALPL* would be highly accelerated because new genome editing technologies (as exemplified by the CRISPR/Cas9 system) are being updated daily.

## 7. ALP as Possible New Tools for Cell Research

### 7.1. Usefulness of Live Staining Kit for Isolating Live ALP-Expressing Cells without Fixation

During a reprogramming protocol, candidate iPSC colonies are generally examined for the expression of pluripotent markers, such as SSEA-4, TRA-1-60, and TRA-1-80, using immunocytochemical analyses. This method, however, is typically used after distinct colonies emerge, probably at 21 days post transduction [126]. ALP expression is discernible in the colonies as early as 14 days post transduction and can be used as a marker for the early identification of iPSCs. Singh et al. [127] employed Alkaline Phosphatase Live Stain Kit (provided from ThermoFisher) to label early intermediates during iPSC generation or clonal populations of ESCs/iPSCs for further selection and expansion.

### 7.2. Usefulness of Ecto-Alkaline Phosphatase-Mediated System to Eliminate ALP-Positive Cells

The incomplete differentiation of human iPSCs poses a serious safety risk owing to their potential tumorigenicity, thus hindering their clinical application. Kuang et al. [128] explored the potential of phospho-D-peptides as novel iPSC-eliminating agents. They reported that overexpression of ALP in iPSCs dephosphorylated phospho-D-peptides into hydrophobic peptides that aggregate and induce cell death. They isolated a peptide candidate, D-3, that selectively and rapidly induced toxicity in iPSCs within 1 h, but had little influence on various non-iPSCs, including primary hepatocytes and iPSC-derived cardiomyocytes. Additionally, D-3 prevented residual iPSC-induced teratoma formation in a mouse tumorigenicity assay. Kuang et al. [128] concluded that D-3 is a low-cost and effective anti-iPSC agent for both laboratory use and the safe clinical application of iPSC-derived cells in regenerative medicine.

## 8. Conclusions

TNSALP is a ubiquitous membrane-bound glycoprotein that catalyzes the hydrolysis of phosphate monoesters at basic pH values. It is highly expressed in juvenile cells, such as stem cells (or precursor cells) and PSCs such as ESCs/iPSCs, and is thus considered as one of the markers for defining cells with stemness properties. Additionally, TNSALP is a useful marker for identifying cancerous states in leukemia and some types of cancers. Furthermore, TNSALP is known to be one of the early markers for reporting the presence of intermediate cells, which are reprogrammed from somatic cells toward juvenile cells after transfection with reprogramming factors (also called “Yamanaka’s factors”).

Unfortunately, little is known about the biological role of TNSALP. Analysis of patients with HPP and KO mice, in which *ALPL* has been specifically mutated, suggests the critical role of TNSALP in bone calcification and the prevention of calcification of skeletal and neuronal systems. In experimental systems, the knockdown of *ALPL* expression reduces cell proliferation and differentiation into neurons or oligodendrocytes in NSCs. The overexpression of TNSALP caused the calcification of skeletal muscle cells and endothelial cells. *Akp2* KO mice models often died from seizures, with surviving animals manifesting dental dysplasia. In some cases, the *Akp2*-null mice exhibited impaired growth, vitamin B6-dependent seizures, impaired bone mineralization and apnea, and died before weaning. Thus, improving the understanding of the role of TNSALP in osteogenesis and neurogenesis is important.

However, the mechanisms of TNSALP pluripotency and differentiation of PSCs remains poorly understood. Recent studies clarified the possibility that TNSALP has multiple functions. According to Liu et al. [100], TNSALP may act as a signal regulator by binding to LRP5/6, which activates a canonical Wnt/β-catenin-dependent increase in somatic cell differentiation to osteogenic lineage cells. Furthermore, activation of this system leads to the activation of TNSALP. Therefore, Wnt/β-catenin system-mediated TNSALP loop may exist in some instances, especially when cells are forced to differentiate into the osteogenic lineage.

Notably, expression of TNSALP can trigger the nuclear localization of β-catenin, which is one of the key regulators accelerating cell differentiation to PSCs. This means that forced expression of *ALPL* in ALP-negative cells might be the first step through which cells are reprogrammed to juvenile PSCs. Supporting this hypothesis, we demonstrated that ALP-positive HDDPCs are more amenable to be reprogrammed into iPSCs than ALP-negative HDDPCs when cells are forced to be reprogrammed by transfection with Yamanaka’s factors [79]. In this regard, it would be interesting to test whether the forced expression of *ALPL* in ALP-negative HDDPCs triggers somatic cell differentiation to iPSCs. These recently developed gene-engineering technologies improve the understanding of the biological roles of TNSALP. 

## Figures and Tables

**Table 1:** Summary ^1^ of the gene nomenclature, accession number, and common names for the human and mouse alkaline phosphatase (ALP).

Genes	Accession Numbers	Protein Names	Tissue Distribution	Function
Human genes *ALPL*	NM_000478	TNAP, TNSALP, L/B/K ALP	Developing nervous system, skeletal tissues, liver and kidney	Skeletal mineralization
*ALPP*	NM_001632	PLAP, PLALP, Regan isoenzyme	Syncytiotrophoblast, a variety of tumors	Unknown
*ALPP2*	NM_031313	GCAP, GCALP, NAGAO isozyme	Testis, malignant trophoblasts, testicular cancer	Unknown
*ALP1*	NM_001631	IAP, IALP, Kasahara isoenzyme	Gut	Fat absorption, Detoxification of lipopolysaccharide
Mouse genes *Akp2 (Alpl)*	NM_007431	TNAP, TNSALP, L/B/K ALP	Developing nervous system, skeletal tissues and kidney	Skeletal mineralization
*Akp3*	NM_007432	IAP, IALP, dIALP	Gut	Fat absorption, detoxification of lipopolysaccharide
*Akp5 (Alpl2)*	NM_007433	EAP	Preimplantation embryo, testis, gut	Early embryogenesis
*Akp-ps1*	NG 001340	ALP pseudogene, pseudoALP	Not described	
*Akp6 (Alpi)*	AK008000	gIALP	Gut	Under investigation

^1^ Created based on the data of Linder et al. [19]. Abbreviations: dIALP, duodenum-specific intestinal ALP; EAP, embryonic ALP; GCALP (or GCAP), germ cell ALP; gIALP, global IALP; IALP (or IAP), intestinal ALP; L/B/K ALP, liver/bone/kidney ALP; PLALP (or PLAP), placental ALP; TNSALP (or TNAP), tissue-nonspecific ALP.

**Table 2:** Summary of knock-out (KO), knock-in (KI) or transgenic (Tg) mice for assessing gene function of alkaline phosphatase (ALP) family.

Methods	Target Gene	Outcome (Note)	Reference
Overexpression	*ALPP*	Tg mice overexpressing human *PLALP* systemically were produced. There were no adverse effects on mouse development or viability.	[20,21]
Gene targeting for KO	*ALP1*	Homozygous mutants (called *Akp3^−/−^*) were histologically normal and fertile. However, accelerated transport of fat droplets through the intestinal epithelium and elevation of serum triglyceride levels were discernible, which was associated with higher intestinal Ca^2+^ uptake.	[22,23]
Overexpression	*ALPP2*	Tg mouse lines harboring human *ALPP2* linked to short or long promoter region of *ALPP2* were produced. A 450 bp promoter sequence directs *GCALP* expression to the intestine and endothelial cells, while a 1.7 kb promoter sequence directs *GCALP* expression to the spermatogenic lineage and to the eight-cell embryos through the blastocysts.	[24]
Gene targeting for KO	*Akp5 (Alpl2)*	No obvious phenotypic abnormalities. Normal reproductive activity with acquisition of live offspring, indicating the nonessential role of EAP during embryonic development.	[25]
Gene targeting for KO	*Akp5 (Alpl2)*	In homozygous mutants (called *EAP.ko*), preimplantation embryos exhibited slower development and higher rates of degeneration, delayed parturition, and reduced litter size.	[26]
Gene targeting for KI	*ALPL*	In homozygous mutants (called *Alpl^tm1Sor^*), primordial germ cells appear unaffected indicating that *ALPL* is not essential for their development or migration. At first, the mice exhibited normal skeletal development; however, homozygous mutant mice developed seizures and apnea at approximately two weeks after birth, and died before weaning. Rescued animals subsequently develop defective dentition. TNSALP modulates T lymphocyte function (specifically T cell-dependent colitis) when examined using heterozygous *Alpl^tm1Sor^* mice.	[27,28,29]
Gene targeting for KO	*ALPL*	In homozygous mutants (called *Alpl^tm1Jlm^* or *Akp2^−/−^*), abnormal bone mineralization was evident. Morphological changes in the osteoblasts, aberrant development of the lumbar nerve roots, disturbances in intestinal physiology, increased apoptosis in the thymus, and abnormal spleens are also discernible. Loss of *ALPL* causes myelin abnormalities and synaptic dysfunction.	[25,30,31]
Gene targeting for KI	*ALPL*	KI of Cre expression unit was carried out into the region between exons 6 and 7 of *ALPL* gene. The resulting line was called *Alpl^tm1(cre)Nagy^*. After crossed *Alpl^tm1(cre)Nagy^* line with the double-reporter line, Z/AP, human ALP expression is discernible in PGCs at E9.5–10.5. After mid-gestation, however, it was also expressed in the labyrinthine region of the placenta, the intestine and the neural tube.	[32]
Gene targeting for KI	*ALPL*	KI of an around 12 kb genomic sequence of *Akp2* in which two *loxP* sites (located in introns 2 and 4, respectively) and a cassette containing neomycin resistance gene expression unit into the endogenous *Akp2* locus. This floxed mouse (called *Alpl^flox/flox^*) is normal in the absence of Cre expression. However, in the presence of Cre, the deletion of exons 3 and 4 should occur, which may result in the ablation of endogenous TNSALP expression.	[33]
Overexpression	*ALPL*	Tg mouse line (called “*Col1a1-Tnap*”) expressing human *ALPL* under control of an osteoblast-specific collagen type I α1 chain (*Col1a1*) promoter was produced. This line is healthy and exhibits increased bone mineralization.	[34]
Overexpression	*ALPL*	Tg mice carrying human *ALPL* under the vascular smooth muscle cell-specific transgelin (*Tagln*) promoter were produced. They developed severe arterial medial calcification and reduced viability.	[35]
Overexpression	*ALPL*	Tg mice (celled “*Endothelial TNSALP mice*”) carrying *ALPL* under the endothelial-specific tunica intima endothelial kinase 2 (*Tie2*) promoter were produced. They survived well into adulthood and displayed generalized arterial calcification together with elevated blood pressure and compensatory left ventricular hypertrophy.	[36]

Abbreviations: *ALPL*, tissue-nonspecific ALP (TNSALP); E, embryonic day; *EAP*, embryonic ALP; *GCALP,* germ cell ALP; PGC, primordial germ cell; *PLALP,* placental ALP.

**Table 3:** Summary of candidate genes whose expression fluctuates after overexpression of exogenous *ALPL* or suppression of endogenous *ALPL* in mammals.

Method ^1^	Type of Cells Used	Genes Upregulated	Genes Downregulated	Genes Unaltered	References
siRNA	High-ALP-expressing cells derived from human osteoblast-like cells (HOS)	*COL1A1* *RUNX2*		*OCN*	[48]
siRNA	3T3-F442A adipocytes	*GAPDH* *ADIPOQ* *FABP4*	*LEP*		[58]
Revamisol	T3-F442A adipocytes		*PPARG* *LEP* *ADIPOQ * *ATGL*	*TIP47* *SCARB1*	[58]
Revamisol	Murine osteoblast precursor cells		*RUNX2* *SP7* *BGLAP2* *DMP1*		[71]
Overexpression	Tg mice overexpressing *ALPL* gene	*OPN* *BMP2 * *MGP* *SOX9* *SLC20A1*	*ACTA2* *TAGLN*	*ANK*	[35]
Overexpression	Dermal papilla	*BMP4* *AXIN2* *VCAN * *LEF1* *SOX2*		*BMP2* *NOG* *FGF7*	[70]

^1^ Suppression of endogenous *ALPL* expression was carried out by transfection with small interfering RNA (siRNA) or by treating cells with revamisol, a reversible inhibitor for TNSALP. Additionally, an increased level of TNSALP was achieved by overexpression of exogenous *ALPL* gene. Then, genes upregulated, downregulated or unaltered after these treatments were examined through comparison with untreated parental cells. Abbreviations: ACTA2, actin α2; ADIPOQ, adiponectin; ALPL, a gene encoding tissue non-specific alkaline phosphatase (TNSALP); ANK, progressive ankylosis protein; ATGL, adipose triglyceride lipase; AXIN2, axin 2; BGLAP2, bone γ-carboxyglutamate protein 2; BMP2, bone morphogenetic protein 2; BMP4, bone morphogenetic protein 4; COL1A1, collagen type I α1 chain; FABP4, fatty acid-binding protein 4; FGF7, fibroblast growth factor 7; GAPDH, glycerophosphate dehydrogenase; LEF1, lymphoid enhancer-binding factor 1; LEP, leptin; MGP, matrix Gla protein; NOG, noggin; OCN, osteocalcin; OPN, osteopontin; PPARG, peroxisome proliferator activated receptor gamma; RUNX2, runt-related transcription factor 2; SCARB1, scavenger receptor class B member 1; SLC20A1, solute carrier family 20 member 1; SP7, Sp7 transcription factor; SOX2, SRY-box transcription factor 2; SOX9, SRY-box transcription factor 9; TAGLN, transgelin; Tg, transgenic; TIP47, tail-interacting protein of 47 kD; VCAN, versican.
